# The Magnitude of Postconvulsive Leukocytosis Mirrors the Severity of Periconvulsive Respiratory Compromise: A Single Center Retrospective Study

**DOI:** 10.3389/fneur.2019.01291

**Published:** 2019-12-06

**Authors:** Jose L. Vega, Prabhu Emmady, Christina Roels, John Conforti, Catalina Ramirez, Mehmet T. Dorak

**Affiliations:** ^1^Department of Neurosciences and Stroke, Novant Health, Forsyth Medical Center, Winston-Salem, NC, United States; ^2^TeleNeurologia SAS, Medellin, Colombia; ^3^Department of Critical Care, Novant Health, Forsyth Medical Center, Winston-Salem, NC, United States; ^4^Seguros Medicos Suramericana, Medellin, Colombia; ^5^School of Life Sciences, Pharmacy & Chemistry, Kingston University London, United Kingdom

**Keywords:** leukocytosis, respiratory failure, generalized epileptic convulsions, pulmonary edema, neurogenic pulmonary edema, catecholamines

## Abstract

**Background:** Generalized epileptic convulsions frequently exhibit transient respiratory symptoms and non-infectious leukocytosis. While these peri-ictal effects appear to arise independently from one another, the possibility that they stem from a common ictal pathophysiological response has yet to be explored. We aimed to investigate whether peri-ictal respiratory symptoms and postictal leukocytosis coexist.

**Methods:** We performed a single center retrospective chart review of 446 patients brought to our emergency department between January 1, 2017 and August 23, 2018 for the care of generalized epileptic convulsions with or without status epilepticus. We included 152 patients who were stratified based on the presence (PeCRC+) or absence (PeCRC–) of overt periconvulsive respiratory compromise (PeCRC). In addition, patients were stratified based on the presence or absence of postconvulsive leukocytosis (PoCL), defined as an initial postconvulsive white blood cell (WBC) count ≥ 11,000 cells/mm^3^. Triage vital signs, and chest x ray (CXR) abnormalities were also examined.

**Results:** Overt PeCRC was observed in 31.6% of patients, 43% of whom required emergent endotracheal intubations. PoCL was observed in 37.5% of patients, and was more likely to occur in PeCRC+ than in PeCRC– patients (79.2 vs. 18.2%; OR = 17.0; 95% CI = 7.2–40.9; *p* < 0.001). Notably, the magnitude of PoCL was proportional to the severity of PeCRC, as the postconvulsive WBC count demonstrated a negative correlation with triage hemoglobin oxygen saturation (R = −0.22; *p* < 0.01; CI = −0.48 to −0.07). Moreover, a receiver operating characteristic analysis of the WBC count's performance as predictor of endotracheal intubation reached a significant area under the curve value of 0.81 (95% CI = 0.71–0.90; *p* < 0.001). Finally, PeCRC+ patients demonstrated frequent CXR abnormalities, and their postconvulsive WBC counts correlated directly with triage heart rate (R = 0.53; *p* < 0.001).

**Conclusion:** Our data support the existence of an ictal pathophysiological response, which induces proportional degrees of PoCL and PeCRC. We suggest this response is at least partially propelled by systemic catecholamines.

## Introduction

Sporadically, epileptic seizures are accompanied by a transient and non-infectious leukocytosis of unclear clinical significance (1). This phenomenon rarely occurs after self-limiting complex partial seizures (7%) (1) but it is common after generalized epileptic convulsions, particularly when they progress into convulsive status epilepticus (63%) (2). These observations suggest that postictal WBC counts might be physiologically linked to the clinical outcomes of different epileptic seizure types. While the specific factors that induce postconvulsive leukocytosis (PoCL) remain undefined, a role for systemic catecholamines is suggested by prior demonstrations that: [1] generalized epileptic convulsions significantly increase systemic epinephrine and norepinephrine levels (3–5) and that [2] systemic injections of catecholamines consistently increase the peripheral white blood cell (WBC) count (6, 7). At a molecular level, catecholamines appear to accomplish this effect by disrupting adhesion sites between WBCs and the endothelium (8). Potential WBC sources for this response include the lymphatic vessels, spleen, bone marrow (7) and lungs (9).

Like PoCL, peri-convulsive respiratory compromise (PeCRC) also occurs across a wide range of epileptic semiologies (10, 11). It typically manifests itself as transient alterations in respiration such as apnea, tachypnea, or hypopnea, coupled with decreased hemoglobin oxygen saturations (SpO_2_) (10, 11). Further, PeCRC often demonstrates acute neurogenic pulmonary edema (12, 13), a potentially lethal condition associated with severe tachycardia and with a shift of intravascular fluid into the perialveolar interstitial space and alveoli (14). Acute neurogenic pulmonary edema has also been associated with postictal leukocytosis (15).

Proposed mechanisms to explain neurogenic pulmonary edema include inflammatory disruption of the pulmonary vascular endothelium, ictal negative intrathoracic pressure, and disruption of the pulmonary endothelial Na/K pump due to prolonged hypoxia (14). In addition, a leading theory suggests that neurogenic pulmonary edema is triggered by a massive increase in systemic catecholamines which causes pulmonary vasoconstriction and disrupts the capillary-alveolar membrane (14, 16). Interestingly, neurogenic pulmonary edema appears to occur more frequently than previously believed (17), and it is common in convulsive status epilepticus (14, 18). Thus, it is possible that an unknown fraction of PeCRC cases relates to the same pathophysiological process which drives neurogenic pulmonary edema. This notion is supported by a prior investigation in which 40% of generalized epileptic convulsions were associated with the development of non-specific chest x ray (CXR) abnormalities akin to mild pulmonary edema (19). In this study we sought to investigate the relationship between PoCL and PeCRC in patients brought to the hospital for emergent care of generalized epileptic convulsions.

## Methods

### Patient Selection and Determination of PeCRC and PoCL

We performed a retrospective chart review at a large tertiary care hospital in North Carolina, United States, with approval from its institutional review board. The study included patients admitted through our emergency department between January 1, 2017 and August 23, 2018, for the treatment of generalized epileptic convulsions with impaired awareness, with or without status epilepticus. For the purposes of this article the word periconvulsive is meant as the time period that lapses between the onset of a generalized epileptic convulsion and the postconvulsive period which precedes a complete return to normal mentation. We generated a patient source list from electronic medical records (EMRs) containing ICD-10 codes R56.9 (seizures, 80.4%) and G40.901 (status epilepticus, 19.6%) (*n* = 446), regardless of whether or not patients carried a pre-existing diagnosis of epilepsy. Only EMRs demonstrating unequivocal written evidence of at least one witnessed generalized epileptic convulsion occurring immediately prior to arrival at the emergency department, or inside the emergency department, were selected for the study. Seventy-three EMRs (i.e., patients) reflecting convulsive events originally deemed to be epileptic but later reclassified as syncope, tremor, or other diagnoses, were excluded from the study. Non-convulsive (*n* = 101) and non-epileptic (*n* = 13) seizure semiologies were also excluded from the study. In addition, EMRs lacking a postconvulsive complete blood count drawn within the first 12 h of admission (CBC1; *n* = 67), exhibiting evidence of recent or active infections (*n* = 26), or the possibility of abnormal immunological function due to disease (e.g., HIV+ status) or due to active treatment with chemotherapy, steroids, or other immunosuppressant agents (*n* = 14), were also excluded. The remaining EMRs (*n* = 152) were separated into two groups depending on periconvulsive respiratory status. The PeCRC+ group included EMRs that contained unequivocal written descriptions of convulsive or postconvulsive respiratory compromise. These descriptions, as provided by witnesses and medical providers, included expressions such as “labored breathing,” “cyanosis,” “became apneic,” “stopped breathing,” “could not breathe,” “was gasping for air,” and “lips turned blue,” among others. Evidence of endotracheal intubation was defined as placement of a tracheal airway followed by a period of mechanical ventilation. The PeCRC– group included EMRs which did not contain such descriptions. EMRs were also stratified according to the magnitude of the initial postconvulsive WBC count (WBC1) obtained from CBC1. As previous publications on this subject have considered postictal leukocytosis as an abnormal elevation of the WBC count (1, 2), WBC1 counts ≥ 11,000 cells/mm^3^, the lowest limit of the abnormal WBC count range at our institution, were included in the PoCL+ group. Patients with WBC1 counts ≤ 10.900 cells/mm^3^ were included in the PoCL– group. Initial postconvulsive hemoglobin, emergency department triage vital signs, percent SpO_2_, and the results of initial chest X rays (CXR) obtained within 36 hours of emergency department triage were also recorded. Hemoglobin levels were considered abnormally low when they failed to reach 13.5 g/dL in men, or 12.2 g/dL in women.

### Confirmation of Transient Leukocytosis

By definition PoCL involves a transient elevation of the WBC count after an epileptic seizure (20). Thus, we controlled for the effect that non-transient postictal WBC count elevations had on our results by performing a re-analysis of our data excluding patients whose WBC1 counts increased on a subsequent CBC obtained within 36 h of CBC1 (CBC2).

### Relative Leukocytosis Index (RLI)

To further examine the relationship between the postconvulsive WBC count and periconvulsive respiratory symptoms, we investigated whether minor postconvulsive WBC count elevations within the boundaries of the normal reference range also correlated with periconvulsive respiratory status. This question was assessed by comparing the frequency with which the WBC count from CBC1 (i.e., WBC1) exceeded the WBC count from CBC2 (WBC2) in PeCRC+ and PeCRC– patients. Because none of the patients demonstrated identical WBC counts in both CBC1 and CBC2, we expected that random fluctuations of the WBC count would give two *equally possible* outcomes: WBC1>WBC2 and WBC2>WBC1. To analyze the data, we used an arbitrary index, the RLI, which was calculated as follows: (WBC1–WBC2)/(WBC1). Using this equation, RLIs > 0 are consistent with the outcome WBC1>WBC2 and support the existence of a relative postconvulsive leukocytosis (RPoCL) response if the WBC1 count < 10.900 cells/mm^3^, or of a frank PoCL response if the WBC1 count ≥ 11,000 cells/mm^3^.

### Effect of Hemodilution on Postconvulsive WBC Counts

Hemodilution from continuous infusion of intravenous fluids in the hospital can theoretically decrease the WBC2 concentration and favor an outcome in which WBC1>WBC2. To control for this factor, we compared the normalized change in WBC count observed between CBC1 and CBC2, with the corresponding normalized change in hematocrit between these two samples. For normalization, the smallest change between CBC1 and CBC2 for each data set was defined as 0%, and the largest change as 100%.

### Quantification of Chest X Ray (CXR) Abnormalities

To investigate the presence of acute CXR abnormalities in our cohort, we reviewed available reports of postconvulsive CXRs acquired no later than 36 h after admission. CXR images were not reviewed. Patients whose CXR reports suggested chronic pulmonary pathology (e.g., pulmonary nodules or masses) or cardiac abnormalities (e.g., cardiomegaly) were excluded from this part of the analysis. Patients met criteria for the CXR– (normal) group if their CXR readings denoted unequivocally normal findings. Such readings included terms such as “normal,” “nothing acute,” and “no acute cardiopulmonary disease.” Patients met criteria for the CXR+ (i.e., abnormal) group if their CXR readings demonstrated any other findings, including opacities, infiltrates, atelectasis, pulmonary edema, vascular congestion, pleural effusions, and increased interstitial markings.

### Data Analysis

Continuous variables were compared via parametric or non-parametric tests based on the presence or absence of a normal distribution, respectively. Normality was tested by D'Agostino and Person's test. Categorical variables were reported as whole numbers and compared via Fishers exact test, or Chi Square test for trend as appropriate. Significant associations between relevant continuous variables were assessed using Pearson correlation. The frequency of RLIs > 0 was tested using a binomial test whose null hypothesis assumed equal outcomes for the relationship between WBC1 and WBC2. Normalized WBC count changes were compared with their corresponding normalized hematocrit changes via two-tailed Wilcoxon matched pairs sign-ranked test. Receiving Operating Characteristic (ROC) curves were generated to summarize the relationship between WBC1 count, and heart rate, with endotracheal intubation. An area under the curve (AUC) value of 0.5 suggests a predictive performance not different from chance. An AUC value of 1.0 suggests a predictive performance with 100% sensitivity and specificity. Statistical significance was defined by *p* < 0.05. Prism version 8.0 was used for data analysis and graph design.

## Results

### Demographic Characteristics of PeCRC+ and PoCL+ Patients

**Figure 1:**
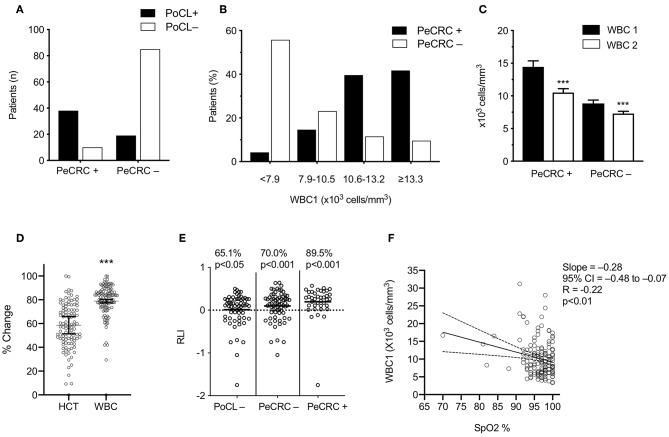
Transient leukocytosis coexists with periconvulsive respiratory pathology. **(A)** PoCL+, WBC1 count ≥ 11 × 10^3^ cells/mm^3^; PoCL–, WBC1 count < 11 × 10^3^ cells/mm^3^; PeCRC+, periconvulsive respiratory compromise. PCRC–, absence of PeCRC; **(B)** The <7.9 bars include the lowest two WBC1 quintiles (range 3.3–31.2 × 10^3^ cells/mm^3^). **(C)** Average WBC1 and WBC2 were drawn within 1.1 ± 1.6 h of triage and within 18.4 ± 9.4 h of WBC1, respectively. **(D)** Normalized percent change between CBC1 and CBC2 for each data set; Statistical analysis via two-tailed Wilcoxon matched pairs sign-ranked test. HCT, hematocrit; WBC, White blood cell count. **(E)** RLI, WBC1–WBC2/WBC1; *p*-values show the significance of a binomial test comparing the observed frequency of RLI > 0 for each group against an expected frequency of 50%; **(F)** SpO_2_, triage percent oxygen saturation measured by pulse oximetry (*N* = 146); analyzed via Pearson correlation. All error bars represent the mean ± s.e.m. ^***^p < 0.001. Graphs C-E stem from the confirmed transient leucocytosis cohort.

Age, gender, triage blood pressure, triage temperature, average blood collection times, average CXR acquisition times, and average hemoglobin levels were similar across all subgroups (Table 1). However, CBC1 hemoglobin levels failed to reach the normal reference range in 39.1% (27/69) of men and in 32.5% (27/83) of the women included in this study. PeCRC+ rates were not affected by hemoglobin levels (low hemoglobin = 31.3%; normal hemoglobin 31.6%; *p* = 0.86; Fishers exact test). Triage heart rate, was significantly higher in PeCRC+ and PoCL+ patients by comparison with PeCRC– and PoCL– patients, respectively. A higher triage heart rate was also seen in INT+ patients by comparison with INT– patients (Table 1). The average time from triage to CBC1 collection was 1.1 ± 1.4 h (Table 1). The WBC1 count was significantly higher in PeCRC+, PoCL+, CXR+, and INT+ patients by comparison with PeCRC–, PoCL–, CXR–, and INT– patients, respectively (Table 1). Forty-eight (31.6%) patients were PeCRC+, and 21 of these (43.8%) required endotracheal intubation (INT+). Accordingly, 131 patients did not require endotracheal intubation (INT–) (Table 1). Relevant clinical aspects of INT+ patients, as documented in their EMRs, are described in Table 2. Fifty-seven patients (37.5%) were PoCL+. PoCL was present in 38 out of 48 PeCRC+ patients (79.2%), but only in 19 out of 104 (18.2%) PeCRC– patients (OR = 17.0; 95% CI = 7.2–40.9; Fishers exact test; *p* < 0.001; Figure 1A; Table 1). In addition, the WBC1 count of PeCRC+ and PeCRC– patients exhibited opposite distributions across the cohort's WBC1 count range, with the majority of PeCRC+ patients falling in the two highest quintiles and the majority of PeCRC– patients falling in the three lowest quintiles (range = 3.3–31.2 cells/mm^3^; quintile cutoff values were 6,200; 7,900; 10,500 and 13,200 cells/mm^3^) (Figure 1B; Table 1).

**Table 1:** Data and statistics summary for the complete patient cohort.

		**PeCRC+**	**PeCRC–**	***p***	**PoCL+**	**PoCL–**	***p***	**CXR+**	**CXR–**	***p***	**INT+**	**INT–**	***p***
*N* (%)	152 (100)	48 (31.6)	104 (68.4)	–	57 (37.5)	95 (62.5)	–	43 (40.2)	64 (59.8)	–	21 (13.8)	131 (86.2)	–
PeCRC+ *N* (%)	48 (31.6)	–	–	–	**38**	**10**	^***^	**25**	**18**	^**^	–	–	–
PeCRC– *N* (%)	104 (68.4)	–	–	–	**19**	**85**		**18**	**46**	^*^^d^	–	–	–
WBC1^a^	10.2 ± 5.0	14.0 ± 5.0	8.4 ± 3.9	^***^^d^	15.2 ± 4.4	7.2 ± 1.9	^***^^d^	12.0 ± 5.2	10.1 ± 4.9	^*^^d^	15.7 ± 6.6	9.3 ± 4.0	^***^^d^
WBC1^a^ <7.9^b^; *N* (%)	60 (39.5)	**2**	**58**	^***^	–	–	–	**8**	**25**	^*^	**1**	**59**	^***^
WBC1^a^ 7.9–10.5; *N* (%)	31 (20.4)	**7**	**24**		–	–	–	**9**	**15**		**3**	**28**	
WBC1^a^ 10.6–13.2 *N* (%)	31 (20.4)	**19**	**12**		–	–	–	**15**	**12**		**7**	**24**	
WBC1^a^ 13.3–31.2 N (%)	30 (19.7)	**20**	**10**		–	–	–	**11**	**12**		**10**	**20**	
Hemoglobin (g/dL)	13.1 ± 1.9	13.1 ± 2.3	13.1 ± 1.6	NS	13.2 ± 2.1	13.0 ± 1.7	NS	12.7 ± 2.1	13.2 ± 1.8	NS	13.4 ± 2.3	13.0 ± 1.8	NS
Hours to WBC1	1.1 ± 1.4	1.2 ± 1.5	1.0 ± 1.4	NS^d^	1.2 ± 1.6	1.0 ± 1.3	NS^d^	0.9 ± 0.8	1.3 ± 1.5	NS^d^	1.1 ± 1.0	1.1 ± 1.5	^***^^d^
CXR+ N (%)^c^	43 (40.2)	–	–	–	**24**	**19**	^*^	–	–		**14**	**29**	^**^
CXR– N (%)^c^	64 (59.8)	–	–	–	**22**	**42**		–	–		**7**	**58**	
Hours to CXR^c^	2.9 ± 4.4	2.5 ± 2.8	3.1 ± 5.2	NS^d^	2.3 ± 2.8	3.4 ± 5.3	NS^d^	3.2 ± 5.0	2.7 ± 4.0	NS^d^	2.5 ± 2.8	3.0 ± 4.8	NS^d^
Age (years)	61.1 ± 18.2	60.5 ± 18.6	61.5 ± 18.1	NS	60.1 ± 17.8	61.8 ± 18.5	NS^d^	64.7 ± 16.8	63.3 ± 16.6	NS	57.7 ± 13.9	61.7 ± 18.7	NS^d^
Male	69 (45.4)	**22**	**47**	NS	**30**	**39**	NS	**18**	**33**	NS	**8**	**61**	NS
Female	83 (54.6)	**26**	**57**		**27**	**56**		**25**	**31**		**13**	**70**	
SBP (mm Hg)	147.7 ± 35.7	147.1 ± 41.81	148 ± 32.8	NS	152 ± 40.21	145 ± 30.7	NS	140.3 ± 39.7	153.1 ± 35.4	NS	152 ± 48.6	147 ± 33.4	NS
DBP (mm Hg)	82.2 ± 20.1	81.9 ± 19.8	82.3 ± 20.3	NS	84.1 ± 22.1	81.0 ± 18.9	NS	77.6 ± 21.1	86.2 ± 21.1	^*^	88.1 ± 22.2	81.2 ± 19.7	^*^
Heart rate (beats per minute)	99.0 ± 26.9	109.3 ± 28.0	94.3 ± 25.1	^***^	110.8 ± 30.2	91.6 ± 22.0	^***^	102.4 ± 27.7	99.7 ± 27.0	NS	111.6 ± 33.6	97.0 ± 25.2	^*^
Temperature (°F)	98.5 ± 1.2	98.5 ± 28.2	98.4 ± 1.0	NS^d^	98.6 ± 1.2	98.4 ± 1.2	NS	98.7 ± 1.8	98.4 ± 1.0	NS^d^	98.7 ± 2.1	98.4 ± 1.0	^*^d

*CXR+ = Abnormal chest X ray; CXR− = Normal chest X ray; DBP = Diastolic blood pressure; INT+ = Intubated; INT− = Not intubated; PeCRC+ = Periconvulsive respiratory compromise; PeCRC− = Absence of periconvulsive respiratory compromise; PoCL+ = WBC1 count < 11,000 cells/mm^3^; PoCL− = WBC1 count < 11.000 cells/mm^3^; SBP = Systolic blood pressure; WBC1 = Initial postictal white blood cell count. Bolded numbers represent discrete 2X2 or 2X4 contingency tables defined by corresponding rows and columns, and analyzed via Fishers exact test or Chi square test for trend, respectively*.

*** <0.001;

** <0.01;

* *<0.05. All continuous variables are expressed as mean ± SD. All comparisons were made via two-tailed unpaired Student t-test unless otherwise indicated*.

a *x10^3^ cells/mm^3^*.

b *Combines the two lowest WBC1 quintiles*.

c *Out of 107 patients whose care included an CXR within 36h of admission*.

d *Comparison via two-tailed Mann Whitney test*.

**Table 2:** Clinical characteristics of intubated patients.

**INT. LOC**.	**INT. CAUSE**	**BENZO^b^**	**AED^b^**	**SpO_**2**_ (%)**	**WBC1^c^**	**WBC2^c^**	**TTE**	**HR**	**CXR^d^**	**EEG^e^**	**DX**	**Possible seizure etiology^f^**
ED	RD	LOR 2 mg	NG	NA	10.5	8.7	16.2	80	Opacity	+	SE	Posterior Reversible Encephalopathy
ED	RD	LOR 4 mg	LEV 1 g	96	10.7	6.1	23.4	96	Atelectasis	–	SE	Brain Metastases
ED	RD, AG	LOR 8 mg	NG	93	11.1	5.6	13.4	110	Atelectasis	–	SE	Old subarachnoid hemorrhage
***AH ED***^a^	***AP***	***LOR 2 mg***	***LEV 1 g***	***NA***	***12.8***	***14.8***	***124.1***	***70***	***Atelectasis***	***–***	**SE**	***Severe hyperglycemia, stroke***
EMS	RD	NG	NG	100	12.8	9.0	16.7	94	Pulmonary edema	–	SE	Unclassified epilepsy, not on AED
AH ED	AG	LOR 5 mg	NG	98	9.3	10.7	7.3	100	Atelectasis	–	GEC	Post-stroke epilepsy
ED	RD	LOR 5 mg	NG	96	13.0	7.1	12.2	50	Atelectasis	–	SE	Intentional overdosed on diphenhydramine
ED	MS, RD, AG	LOR 8 mg	NG	92	22.0	17.2	17.8	107	Interstitial markings	–	GEC	Unclassified epilepsy
ED	RD	LOR 2 mg	NG	97	21.9	16.2	16.6	72	Normal	–	SE	Unclassified epilepsy
ED	MS, AP	LOR 2 mg	LEV 1 g	91	31.2	16.2	24.8	174	Atelectasis	–	SE	Idiopathic epilepsy, polysubstance abuse
ED	RD	NG	NG	100	5.0	14.0	62.5	69	Pleural effusion	–	GEC	New intracranial hemorrhage
ED	AP	NG	NG	98	11.7	11.6	8.0	118	Atelectasis	+	GEC	Post-stroke epilepsy
ED	AP	LOR 2 mg	NG	98	21.9	11.3	7.6	136	Normal	–	SE	Unclassified epilepsy
***ED***^a^	***IR***	***LOR 2mg***	***NG***	***93***	***9.4***	***6.8***	***20.2***	***110***	***Normal***	***–***	**GEC**	***New meningitis***
ED	RD	LOR 6 mg	NG	84	16.5	16.2	32.6	156	Atelectasis	–	SE	Opiate overdose
ED	RD, AG	LOR 4 mg	NG	97	11.9	8.8	12.6	126	Normal	–	GEC	Post-stroke epilepsy
ED	MS, AP	LOR 2 mg	NG	91	22.8	16.1	40.6	165	Pulmonary edema	–	SE	Idiopathic epilepsy
ED	AB	LOR 2 mg	NG	98	28.0	19.1	14.2	142	Normal	–	SE	Idiopathic epilepsy, medication non-compliance
ED	RA	LOR 2 mg	NG	91	15.4	13.7	TRACH	146	Normal	–	GEC	Cerebral palsy
ED	RD	MID 10 mg	LEV 1 g	95	15.2	12.6	22.8	122	Normal	–	SE	Cerebritis
***AH ED***^a^	***RD***	***LOR 2 mg***	***NG***	***100***	***16.1***	***17.9***	***5.5***	***100***	***Atelectasis***	***–***	**GEC**	***Cocaine***

*AB, Agonal breathing; AED, Antiepileptic medications; AG, Agitation; AH, Affiliated hospital; AP, Airway protection; BENZO, benzodiazepine; CXR, Chest X ray; DX, Diagnosis; ED, Emergency department; EEG, electroencephalogram; EEG+, Seizures on EEG; EEG– No seizures on EEG; EMS, Emergency medical services; GEC, Generalized epileptic convulsion; HR, Heart rate (beats per minute) recorded at ED triage; INT = Intubation; INT LOC, Intubation location; IR, Imaging requirement; LEV, Levetiracetam; LOR, Lorazepam; MID, Midazolam; MS, Multiple seizures; NA, Not available; NG, None given; RA, Respiratory arrest; RD, Respiratory distress; SpO_2_, Percent oxygen saturation measured by pulse oximetry at ED triage; SE, Status epilepticus; TRACH, tracheostomy; TTE, Time to extubation (hours); WBC1, Initial postictal WBC count obtained within 12 h of admission; WBC2, Second postictal WBC obtained within 36 h of WBC1. Bolded patients were excluded from the confirmed transient leukocytosis reanalysis*.

a *Grayed, italicized rows represent patients excluded from the confirmatory analysis*.

b *Total doses given prior to endotracheal intubation*.

c *× 10^3^ cells/mm^3^*.

d *Abnormality reported by reading radiologist*.

e *+ = seizures; – no seizures, on EEG*.

f *Inferred from EMR notes and imaging*.

### Confirmation of Transient Leukocytosis

Of the 152 original EMRs selected for the study, only 107 contained the results of CBC2s collected within 36 h of CBC1. Average time between CBC1 and CBC2 collection was 18.4 ± 9.4 h. Two patients from the PoCL+, PeCRC+, and INT+ groups who demonstrated WBC2 counts that exceeded their corresponding WBC1 counts were excluded from this part of the analysis. One patient was excluded from the INT+ subgroup because his intubation was performed electively, to facilitate imaging (Table 2). Conclusions and statistical differences reached from the original 152 patients (i.e., conclusions drawn from WBC1 alone; Table 1) were validated in the confirmed leukocytosis analysis, except for the slightly increased diastolic blood pressure of CXR– patients, and the minimally elevated diastolic blood pressure and temperature of INT+ patients (Table 3). While we are unable to explain the blood pressure differences between these subgroups, we believe the elevated temperature seen in the original cohort could stem from undetectable infections in excluded patients.

**Table 3:** Data and statistics summary for the confirmed transient PoCL patient subgroup.

		**PeCRC+**	**PeCRC–**	***p***	**PoCL+**	**PoCL–**	***p***	**CXR+**	**CXR–**	***p***	**INT+**	**INT–**	***p***
*N* (%)	105 (100)	36	69	–	42	63		35	45		18	87	
PeCRC+ *N* (%)	36 (33.9)	–	–	–	**29**	**7**	^***^	**21**	**14**	^**^	–	–	N/A
PeCRC– *N* (%)	69 (66.1)	–	–	–	**13**	**56**		**12**	**31**		–	–	
WBC1^a^	10.2 ± 5.0	14.4 ± 5.5	8.8 ± 4.3	^***^^d^	15.9 ± 4.9	7.3 ± 1.9	^***^^d^	12.3 ± 5.4	11.1 ± 5.3	NS^d^	16.2 ± 7.0	9.6 ± 4.3	^***^^d^
Hours to WBC1	1.1 ± 1.6	1.2 ± 1.7	1.1 ± 1.6	NS^d^	1.3 ± 1.8	1.0 ± 1.5	NS^d^	0.9 ± 0.8	1.4 ± 1.8	NS^d^	1.1 ± 1.1	1.1 ± 1.7	NS^d^
WBC2^a^	8.4 ± 3.5	10.5 ± 3.6	7.3 ± 2.9	^***^^d^	10.6 ± 3.7	6.9 ± 2.4	^***^^d^	9.6 ± 3.9	8.2 ± 3.7	NS^d^	12.2 ± 4.1	7.6 ± 2.8	^***^^d^
Hours from WBC1 to WBC2	18.4 ± 9.4	17.7 ± 9.7	18.7 ± 9.4	NS^d^	20.2 ± 10.4	17.1 ± 8.6	NS^d^	19.2 ± 9.6	16.4 ± 8.7	NS	15.9 ± 11.1	18.9 ± 9.1	NS^d^
WBC1^a^ <7.9^b^; *N* (%)	36 (34.3)	**1**	**36**	^***^	0	36	N/A	**6**	**14**	NS	**1**	**35**	^***^
WBC1^a^ 7.9–10.5; *N* (%)	24 (22.9)	**5**	**18**		0	24		**6**	**11**		**3**	**22**	
WBC1^a^ 10.6–13.2; *N* (%)	20 (19.0)	**15**	**6**		18	3		**14**	**8**		**5**	**15**	
WBC1^a^ 13.3–31.2; *N* (%)	25 (23.8)	**15**	**9**		24	0		**9**	**12**		**9**	**15**	
Hemoglobin (g/dL)	13.1 ± 1.8	13.1 ± 2.1	13.1 ± 1.7	NS	13.3 ± 1.9	13.0 ± 1.9	NS	12.7 ± 2.0	13.4 ± 1.9	NS	13.1 ± 2.2	13.1 ± 1.8	NS
CXR+ *N* (%)^c^	35 (43.7)	–	–	–	**19**	**14**	NS	–	–	–	**12**	**21**	^*^
CXR– *N* (%)^c^	45 (56.3)	–	–		**19**	**26**		–	–		**6**	**39**	
Hours to CXR^c^	3.1 ± 4.9	2.2 ± 2.4	3.7 ± 6.2	^*^d	2.0 ± 2.4^d^	4.0 ± 6.3^d^	^*^d	3.7 ± 5.4	3.1 ± 4.9	NS^d^	1.8 ± 1.4^d^	3.4 ± 5.5^d^	NS^d^
Age (years)	61.1 ± 18.2	60.9 ± 17.8	60.5 ± 16.9	NS	61.3 ± 16.9	60.1 ± 17.4	NS	64.7 ± 17.3	60.6 ± 16.2	NS	58.6 ± 13.2	61.0 ± 17.9	NS
Male *N* (%)	47 (44.8)	**15**	**32**	NS	**21**	**26**	NS	**14**	**23**	NS	**7**	**40**	NS
Female *N* (%)	58 (55.2)	**21**	**37**		**21**	**37**		**22**	**22**		**11**	**47**	
SBP (mm Hg)	147.7 ± 35.7	146.3 ± 44.0	146.5 ± 34.9	NS	153.0 ± 41.8	142.5 ± 35.02	NS	143.3 ± 42.0	147.7 ± 35.2	NS	153.9 ± 51.4	145.2 ± 34.9	NS
DBP (mm Hg)	82.2 ± 20.1	82.9 ± 20.6	82.1 ± 32.3	NS	85.9 ± 23.1	80.0 ± 20.5	NS	79.1 ± 21.9	85.8 ± 22.0	NS	89.9 ± 23.4	80.8 ± 21.1	NS
HR (beats per minute)	98.9 ± 26.9	114.8 ± 28.4	96.0 ± 27.1	^***^	115.1 ± 31.7	93.9 ± 23.8	^***^	105.3 ± 29.0	102.9 ± 28.3	NS	114.6 ± 34.7	99.9 ± 27.0	^*^
Temperature (°F)	98.4 ± 1.2	98.6 ± 1.7	98.6 ± 1.2	NS^d^	98.6 ± 1.3	98.5 ± 1.5	NS	98.8 ± 1.9	98.4 ± 1.1	NS^d^	98.6 ± 2.2	98.5 ± 1.2	NS^d^

CXR+ = Abnormal chest X ray; CXR− = Normal chest X ray; DBP = Diastolic blood pressure; INT+ = Intubated; INT− = Not intubated; PeCRC+ = Periconvulsive respiratory compromise; PeCRC− = Absence of periconvulsive respiratory compromise; PoCL+ = WBC1 count < 11,000 cells/mm^3^; PoCL− = WBC1 count < 11.000 cells/mm^3^; SBP = Systolic blood pressure; WBC1 = Initial postictal white blood cell count; WBC2 = Second WBC measured within 36h of WBC1. Bolded numbers represent discrete 2X2 or 2X4 contingency tables defined by corresponding rows and columns, and analyzed via Fishers exact test, or Chi square test for trend, respectively; p values

*** <0.001;

** <0.01;

* *<0.05; All continuous variables are reported as mean SD. All comparisons were made via two tailed unpaired Student t-test unless indicated otherwise*.

a *x 10^3^ cells/mm^3^*.

b *Combines the two lowest WBC1 quintiles*.

c *Out of 80 patients whose care included a CXR obtained within 36h of admission*.

d *Compared via two-tailed Mann Whitney test*.

### PoCL Occurs Most Frequently in PeCRC+ Patients

The WBC1 count of PeCRC+ patients averaged 14.4 ± 5.5 × 10^3^ cells/mm^3^ (Figure 1C) and, as expected, demonstrated a rapid return to the normal reference range (within 17.7 ± 9.7 h of CBC1; Figure 1C; Table 3). By contrast, while the PeCRC– group demonstrated WBC1 counts that remained within the normal reference range, averaging 8.8 ± 4.3 × 10^3^ cells/mm^3^, this group also experienced a small but significant WBC count drop within a similar time frame (within 18.7 ± 9.4 h of CBC1; Figure 1C; Table 3). These results suggest that a relative leukocytosis also occurred in patients whose WBC1 counts fell within the normal reference range. It should be noted that decreases in WBC count between CBC1 and CBC2 appear to be unrelated to hemodilution, as they significantly exceeded corresponding decreases in hematocrit (Figure 1D).

### The Majority of Generalized Epileptic Convulsions Treated in the Emergency Department Demonstrated Relative Elevations of the WBC Count

To avoid selection bias, all 107 patients whose EMRs contained CBC2s collected within 36 h of CBC1 were used for RLI computations (see below). A large majority of these patients demonstrated RLIs > 0 (Figure 1E), including patients in the PoCL– group, whose WBC1 counts by definition fell within the normal WBC count reference range (65.1% *p* < 0.001; Figure 1E). These results indicate that aside from inducing large PoCL+ responses, generalized epileptic convulsions frequently induce small WBC count elevations which remain within the normal reference range (i.e., RPoCL). If these RPoCL responses are accounted for, PeCRC+ patients demonstrate RLIs > 0 a notable 89.5% of the time (Figure 1E).

### PeCRC Severity Correlates With the Degree of Triage Heart Rate and WBC1 Count Elevations

**Figure 2:**
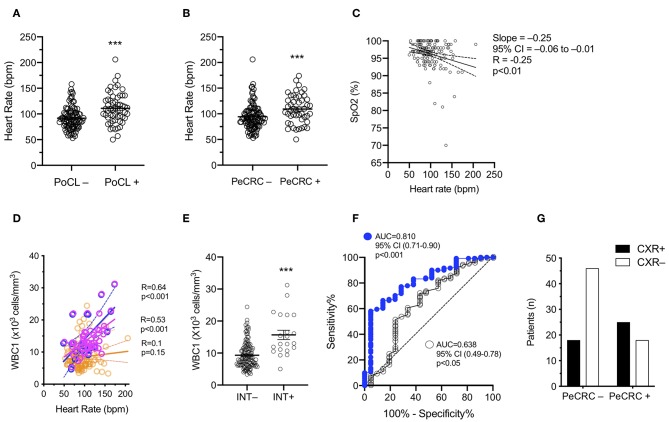
Leukocyte and heart rate elevations mirror periconvulsive respiratory pathology. **(A)** PoCL–, WBC1 count < 11 × 10^3^ cells/mm^3^; PoCL+, WBC1 count ≥ 11 × 10^3^ cells/mm^3^; bpm, beats per minute. **(B)** PeCRC–, Absence of periconvulsive respiratory compromise; PeCRC+, Presence of PeCRC. **(C)** SpO_2_, Triage percent oxygen saturation measured by pulse oximetry (*N* = 146); Analyzed via Pearson correlation. **(D)** Orange circles, PeCRC– (*N* = 104); Pink circles, PeCRC+ (*N* = 48); Blue circles, PeCRC+ INT+ (*N* = 21); Analysis via Pearson correlation test. **(E)** INT–, Not intubated; INT+, Intubated; Analysis via Mann-Whitney test. Error bars show mean ± s.e.m. **(F)** CXR+, abnormal chest x ray; CXR–, normal chest x-ray; OR = 3.5; 95% CI = 1.5–8.0; *p* < 0.01; Fishers exact test. **(G)** AUC obtained from ROC analysis of WBC1 (blue circles) and heart rate (empty circles) performance as predictors of endotracheal intubation (i.e., respiratory failure). ^***^p < 0.001. All graphs represent data obtained from the original GEC cohort.

PoCL+ and PeCRC+ patients demonstrated increased triage heart rates by comparison with PoCL– and PeCRC– patients (Figures 2A,B; Tables 1, 3). In addition the WBC1 count (Figure 1E) and the triage heart rate (Figure 2C; N = 146 as six patients lacked triage SpO_2_ data) correlated inversely with triage SpO_2_. Notably, this correlation could be detected despite the widespread delivery of supplemental oxygen to postconvulsive patients during transport to the emergency department. Supplemental oxygen data were not available for every patient, precluding us from drawing further conclusions about this aspect of our study. Triage heart rate correlated with the WBC1 count of PeCRC+ patients (pink circles in Figure 2D), but not with that of PeCRC– patients (orange circles in Figure 2D). This correlation was particularly significant among INT+ patients (blue circles in Figure 2D), who additionally demonstrated augmented WBC1 counts by comparison with INT– patients (Figure 2E). Moreover, the performance of WBC1 as a predictor of periconvulsive endotracheal intubation analyzed via ROC yielded an AUC of 0.81 (95% CI = 0.71–0.90; *p* < 0.001; Figure 2G) with a maximal Youden index J specificity and sensitivity at a WBC1 cutoff value of 9,200 cells/mm^3^ (sensitivity 58.02%; specificity 95.24%). A similar analysis of the relationship between heart rate and intubation yielded an AUC of 0.64 (Figure 2F).

### PeCRC+ Patients Frequently Exhibit Non-specific CXR Abnormalities

A total of 107 patients from the initial cohort, and 80 patients from the confirmed leukocytosis subgroup had CXRs performed within the first 36 h of admission. The average time between triage and CXR acquisition for each of these groups was 2.9 ± 4.4 and 3.1 ± 4.9 h, respectively. No significant differences in CXR acquisition times were detected in subgroup comparisons, except for CXRs obtained faster for PeCRC+ and PoCL+ patients than for PeCRC– and PoCL– patients in the confirmed leukocytosis group (*p* < 0.05; Table 3). We attribute this result to the urgency of with which physicians order CXRs for patients who exhibit overt respiratory symptoms. PeCRC+ and PoCL+ patients demonstrated more frequent acute, though non-specific, CXR abnormalities by comparison with PeCRC– and PoCL– patients (*p* < 0.01 and *p* < 0.05, respectively; Figure 2G; Tables 1, 3). Fifty-eight CXR abnormalities were seen in the entire cohort. These included opacities (*n* = 5), infiltrates (*n* = 13), atelectasis (*n* = 21), pulmonary edema (*n* = 6), vascular congestion (*n* = 6), pleural effusions (*n* = 5), and increased interstitial markings (*n* = 2).

## Discussion

To our knowledge, this is the first direct investigation of the relationship between PoCL and PeCRC. Our main finding is that, in our patient population, the magnitude of the initial postconvulsive WBC count parallels the severity of periconvulsive respiratory abnormalities. This was evidenced by: [1] a high frequency of PoCL in patients who exhibited PeCRC, and a low frequency of PoCL in patients who did not exhibit respiratory compromise; [2] an inverse correlation between the postconvulsive WBC count and the triage SpO_2_; [3] a ROC analysis of the WBC1 count's performance as a predictor of endotracheal intubation that yielded an AUC of 0.81; and [4] a high frequency of non-specific CXR abnormalities in PoCL+ and PeCRC+ patients. While prior reports had documented leukocytosis in a majority of patients diagnosed with convulsive status epilepticus (2, 21), those reports did not examine the relationship between patients' postconvulsive WBC counts and their periconvulsive respiratory symptoms. Therefore, our work serves as the first direct evidence of a pathophysiological link between these two seemingly unrelated periconvulsive epiphenomena.

Surprisingly, the majority of patients included in this study demonstrated transient postconvulsive WBC count elevations, regardless of their periconvulsive respiratory status. However, the scale of these elevations was proportional to associated respiratory symptoms and was highest, and most frequent, in the PeCRC+ group. Along with the inverse correlation between the initial postconvulsive WBC count and the triage SpO_2_, this result points to the existence of a pathological gradient by which the magnitude of the postconvulsive WBC count mirrors the severity of periconvulsive respiratory symptoms. This notion is best exemplified by two observations from this study: Only one out of the 75 (1.3%) patients in the entire cohort whose postconvulsive WBC counts failed to reach 9,000 cells/mm^3^ required endotracheal intubation. By contrast six out of the seven (85.7%) patients in the entire cohort whose WBC1 counts exceeded 21,000 cells/mm^3^ required endotracheal intubation. As the triage heart rate paralleled the initial postconvulsive WBC count exclusively in PeCRC+ patients, it is reasonable to hypothesize that both WBC count elevations and periconvulsive respiratory symptoms resulted from a common pathophysiological response driven, at least in part, by systemic catecholamines. Such a response might resemble that which has been partially attributed to catecholamines in neurogenic pulmonary edema (14, 18), namely [1] a net augmentation of the amount of blood flowing through the pulmonary blood vessels—either because of an increase in heart rate and cardiac output, or because of a temporary hindrance of the left ventricular function—and [2] a simultaneous vasoconstrictive effect on pulmonary blood vessels. Together, these catecholaminergic effects could increase capillary pressure and precipitate transvascular fluid filtration into the capillary-alveolar membrane and alveoli (14, 18, 22). As neurogenic pulmonary edema has been associated with both, WBC count elevations and significant CXR abnormalities (14, 15, 18, 23), it is also possible that the high frequency of CXR abnormalities observed in the PeCRC+ group could be at least partially induced by a similar combination of catecholaminergic effects. However, despite the resemblance of these results with those of an earlier report of frequent postictal CXR abnormalities following generalized epileptic convulsions (19), the diverse CXR findings reported by our reading radiologists, and the generally poor sensitivity and specificity of roentgenography prevent us from drawing concrete conclusions about the etiology of these imaging results. Consequently, the pathophysiological significance of the non-specific postconvulsive CXR abnormalities unveiled by our work awaits further investigation.

While the specific mechanisms responsible for PoCL in humans have not been thoroughly investigated, systemic catecholamines are likely to play a significant role. This is suggested by consistent reports that [1] generalized epileptic convulsions result in major increases in systemic catecholamines (3–5) and that [2] exogenous catecholamines induce transient elevations of the WBC count (24, 25). In addition, some evidence suggests that catecholamine-induced WBC count elevations result from their boosting effects on heart rate and cardiac output. For instance, a prior investigation of healthy patients found a direct correlation between resting heart rate and peripheral WBC count (26). Similarly, the increased cardiac output that results from prolonged exhalations, or from deep inhalations against a closed glottis (i.e., the Muller maneuver) is accompanied by a rapid elevation of the peripheral WBC count (27). Comparable elevations in the peripheral WBC count have been induced in experimental animals by boosting the cardiac output (28). As the number of marginated leukocytes which resides inside the pulmonary capillary network more than doubles that of all other organs combined (29), some investigators have proposed that transient WBC count elevations seen during states of increased cardiac output are a direct result of augmented pulmonary blood flow (28). In the aggregate, these data suggest that systemic catecholamines play at least a partial role in the induction of both, PoCL and PeCRC.

In light of the above data, the etiology of respiratory abnormalities treated by endotracheal intubation in the periconvulsive period deserves reexamination. Typically, generalized epileptic convulsions result in endotracheal intubation when a high risk of aspiration of oropharyngeal contents, of accidental airway obstruction, or of respiratory suppression from aggressive antiepileptic medication doses is considered imminent (i.e., airway protection) (30). However, as aspiration pneumonia rarely occurs in the postconvulsive period (31), and as respiratory suppression is less likely to occur in generalized epileptic convulsions treated with benzodiazepines than in those treated with placebo (32), the underlying cause of the respiratory symptoms that triggers these endotracheal intubations remains unexplained. In our study, only two of 18 intubations in the confirmed PoCL group intended to protect the airway. Of the remaining sixteen, five of the six patients with the highest postconvulsive WBC counts (range 21.9–31.2 cells/mm^3^) received a total of 2 mg of lorazepam or less prior to intubation. As this low benzodiazepine dose is unlikely to have suppressed their respiratory drives, respiratory symptoms in these patients could stem from alternative causes, such as prolonged central apnea from inefficient contractions of respiratory muscles (e.g., diaphragm, intercostals, etc.). However, two prior investigations found no correlation between convulsive duration and postconvulsive WBC counts (20, 33), suggesting that the duration of a convulsion alone cannot explain the coexistence of PoCL and PeCRC in our patients. We hypothesize that a significant fraction of the endotracheal intubations performed in our patients addressed the pulmonary effects of a cardiopulmonary process which simultaneously causes both PeCRC and PoCL. We further hypothesize that this process is at least partially driven by systemic catecholamines.

The results of this investigation are consistent with previously published rates of periconvulsive respiratory anomalies (34), postictal leukocytosis (1, 2), periconvulsive endotracheal intubations (30), and postconvulsive CXR findings (19). Moreover, our results revealed a high incidence of anemia in our study population, a finding reminiscent of a previously reported negative correlation between the frequency of epileptic seizures and hemoglobin levels (35). However, our conclusions are limited by several factors. First, our study was focused on generalized epileptic convulsions severe enough to require emergent medical attention, and therefore our conclusions cannot be generalized to the overwhelming majority of epileptic convulsions. Second, our inclusion criteria for the PeCRC+ group could have been affected by subjective interpretations of patients' respiratory signs and symptoms, and by unintentional omissions of these interpretations from patients' charts. Third, our data cannot be used to discriminate central, peripheral, ictal, and postictal causes of respiratory compromise, which limits our ability to determine the actual causes of PeCRC in our cohort. Fourth, while the RLI suggests that WBC count elevations of different degrees are the norm following generalized epileptic convulsions, this indirect measure of relative leukocytosis assumes random fluctuations of the WBC count between the first and the second CBCs drawn during a patient's hospital stay. However, despite concurrent hematocrit data suggesting that WBC count reductions between these two CBCs occurred independently of dilutional causes, the WBC count could theoretically also be affected by blood draws, medications, undetectable infections, and other unanticipated causes. Finally, it is possible that a fraction of the CXR abnormalities observed in this study were induced by undiagnosed aspiration pneumonia. However, the fleeting nature of accompanying leukocytosis, and the infrequent incidence of aspiration pneumonia following generalized epileptic convulsions (31) make this an unlikely possibility.

Despite these limitations, and irrespective of whether the pathophysiological mechanisms we have proposed are correct, the relationship between the postconvulsive WBC count and the severity of periconvulsive respiratory symptoms demonstrated by our work deserves further exploration. On a practical level, our results suggest that the postconvulsive WBC count could be used as a marker for the pathophysiological process, or processes, by which generalized epileptic convulsions induce respiratory symptoms in the periconvulsive period. As such, the postconvulsive WBC count has the potential to become an important factor in the search for the mechanisms by which generalized epileptic convulsions result in sudden unexpected death in epilepsy (SUDEP) as the overwhelming majority of SUDEP cases demonstrate PeCRC prior to death, and SUDEP autopsies frequently reveal pulmonary edema, and/or pulmonary hemorrhages (36).

## Conclusion

Our work shows, for the first time, that the magnitude of postconvulsive WBC count elevations corresponds to the severity of periconvulsive respiratory symptoms. As such, the postconvulsive WBC count should be considered as a possible marker for the pathophysiological mechanisms by which respiration is impaired during generalized epileptic convulsions.

## Data Availability Statement

The authors declare that all data used for the preparation of this manuscript can be shared with reviewers and qualified scientists in a deidentified format upon request.

## Ethics Statement

The studies involving human participants were reviewed and approved by Forsyth Medical Center Institutional Review Board. Written informed consent for participation was not required for this study in accordance with the national legislation and the institutional requirements.

## Author Contributions

JV conceived the study, drafted the manuscript, and performed statistical analyses. PE and CRo performed all data collection and reviewed the manuscript. CRa assisted with literature search and reviewed the manuscript. JC provided expert opinion related to pulmonary critical care expertise and reviewed the manuscript. MD performed statistical analyses and reviewed the manuscript.

### Conflict of Interest

JV is the founder of a small Telemedicine startup, TeleNeurologia SAS in Medellin, Colombia. CRa is employed by Seguros Medicos Suramericana in Medellin, Colombia. The remaining authors declare that the research was conducted in the absence of any commercial or financial relationships that could be construed as a potential conflict of interest.
